# Mastering your fellowship: Part 2, 2026

**DOI:** 10.4102/safp.v68i1.6246

**Published:** 2026-03-31

**Authors:** Mergan Naidoo, Klaus von Pressentin, John M. Musonda, Selvandran Rangiah, Ramprakash Kaswa

**Affiliations:** 1 Department of Family Medicine, College of Health Sciences, University of KwaZulu-Natal, Durban, South Africa; 2 Division of Family Medicine, Department of Family, Community and Emergency Care, Faculty of Health Sciences, University of Cape Town, Cape Town, South Africa; 3 Department of Family Medicine, Faculty of Medicine, University of the Witwatersrand, Johannesburg, South Africa; 4 Department of Family Medicine, Faculty of Health Sciences, Walter Sisulu University, Mthatha, South Africa

**Keywords:** family physicians, FCFP (SA) examination, family medicine registrars, postgraduate training, national exit examination, paediatrics, inpatient care

## Abstract

The series ‘Mastering your Fellowship’ provides examples of the question formats encountered in the written and clinical examinations, Part A of the FCFP (SA) examination. The series aims to help family medicine registrars (and supervisors) prepare for this examination. Model answers are available online.

This section in the *South African Family Practice* journal aims to help registrars prepare for the FCFP (SA) examination (Fellowship of the College of Family Physicians). It will provide examples of the question formats encountered in the written exam: Multiple choice questions (MCQs) in the form of single best answer (SBA – Type A) and extended matching questions (EMQ – Type R); short answer questions (SAQ), questions based on the Critical Reading of a Journal article (CRJ: evidence-based medicine) and an example of an Objectively Structured Clinical Examination (OSCE) question. Each of these question types is presented based on the College of Family Physicians blueprint and the key learning outcomes of the FCFP (SA) programme. The MCQs draw on the 10 clinical domains of family medicine, the SAQs align with the five national unit standards and the critical reading section includes evidence-based medicine and primary care research methods.

This edition is based on entrustable professional activity (EPA) 5 (Managing children requiring inpatient care and procedures). We suggest that you attempt to answer the questions (either by yourself or with peers and supervisors) before finding the model answers online: http://www.safpj.co.za/.

Please visit the Colleges of Medicine website for guidelines on the Fellowship examination: https://cmsa.co.za/fellowship-of-the-college-of-family-physicians-of-south-africa-fcfpsa/. We are keen to hear how this series helps registrars and their supervisors prepare for the FCFP (SA) examination. Please email us your feedback and suggestions.

## Extended question

### Instructions: Select the single most appropriate option for each scenario from the list provided (a–i). Each option may be used once, multiple times or not at all

#### Scenario 1:

A 2-year-old baby presents with bilateral pedal oedema, sparse hair and weight for height < −3SD (−3 standard deviations). He has had profuse watery diarrhoea and lethargy for 2 days. On examination, he has sunken eyes, delayed capillary refill of 4 s, a weak radial pulse and cold extremities.

#### Scenario 2:

A 3-year-old child presents with bilateral pedal oedema, sparse hair and weight for height < −3 Z score. He has had profuse watery diarrhoea and lethargy for 1 day. On examination, he has sunken eyes, delayed capillary refill of 2 s and a moderately weak radial pulse.

**Options:**

- ORS (ReSoMal) orally or via NG tube
- IV Ringer’s lactate bolus
- IV 5% dextrose bolus
- IV Half-strength Darrow’s solution with 5% dextrose
- IV albumin
- Maintenance IV fluids 24 h
- Plain water orally
- IV normal saline bolus
- Ongoing breastfeeding

Answer:

Scenario 1: d.

Scenario 2: a.

## Discussion

The approach to treating dehydration in severe acute malnutrition (SAM) involves using ReSoMal (rehydration solution for malnutrition) via nasogastric or oral rehydration solution (ORS) and avoiding rapid IV fluids unless there is a shock with life-threatening features. The ORS (ReSoMal) is designed to have low sodium and high potassium levels and is administered orally or via a nasogastric tube. Rapid IV fluids are not tolerated in children with SAM because of the risk of heart failure and fluid overload.

In scenario 1, we are dealing with a SAM child with kwashiorkor who presents with the WHO definition of shock (capillary refill time of more than 3 s, plus a weak and fast pulse and cool peripheries). Children under 5 years of age with SAM and signs of shock or severe dehydration and who cannot be rehydrated orally or by nasogastric tube should be treated with intravenous fluids, either half-strength Darrow’s solution with 5% dextrose or Ringer’s lactate solution with 5% dextrose. If neither is available, 0.45% saline + 5% dextrose should be used.

In scenario 2, the SAM child with kwashiorkor presents with similar features as in scenario 1 but does not have features of shock or life-threatening conditions. This justifies the use of ReSoMal via nasogastric or ORS.

Further reading

- WHO. Pocketbook of hospital care for children: Guidelines for the management of common childhood illnesses. 2024, World Health Organisation Press, Geneva.
- WHO. Fluid management in severely malnourished children under 5 years of age with shock, World Health Organization, viewed 01 October 2025, from: https://www.who.int/tools/elena/interventions/shock-sam
- South African Department of Health. Paediatric standard treatment guidelines and essential medicines list 2023. Final edition. Pretoria; 2023, South African National Department of Health, Pretoria.
- South African Department of Health. Integrated Management of Childhood Illness 2022. Integrated Management of Childhood Illness Chart Booklet. Pretoria; 2022.
- South African Department of Health. Integrated Management of Children with Acute Malnutrition (IMC-SAM) – National IMC-SAM operational guidance for inpatient and outpatient management for South African hospitals and primary health care facilities. Pretoria; 2015.
- Obonyo N, Maitland K. Fluid management of shock in severe malnutrition: what is the evidence for current guidelines and what lessons have been learned from clinical studies and trials in other pediatric populations? Food Nutr Bull. 2014;35(2_suppl1):S71–S78. https://doi.org/10.1177/15648265140352S111

Short answer:

The family physician’s role is as an ethical and professional decision-maker in the clinical domain of child health, a capacity builder and a consultant.

A 12-year-old girl presents with painful swelling in the deltoid region of her left arm for the past 2 weeks. The coronavirus disease 2019 (COVID-19) vaccine was administered in the same arm 3 months earlier at school. Her parents believe this swelling is related to the vaccine. She has taken pain medication from the local clinic with no relief from her symptoms.

1. As a patient-centred family doctor, how would you respond to the parents’ concerns about the vaccine? (4 marks)
2. Research involving children is crucial to detect possible effects of medical interventions. What are the requirements set by the research ethics committee for enrolling children in a study? (5 marks)
3. The child was brought to your clinic due to swelling in the left arm. Further assessment and X-ray examination revealed a probable bone tumour in the proximal humerus, which was confirmed as an osteosarcoma by histological report. How would you approach delivering the bad news to the child’s family? (6 marks)
4. The orthopaedic surgeon has advised amputation of the arm to prevent metastasis. The child’s parents refuse the amputation. Use the four ethical principles to describe the ethical dilemma(s) arising in this case. (4 marks)
5. How will you address this ethical dilemma in the best interest of the child? Describe your step-by-step approach. (5 marks)

Total: 25 marks

Suggested answers (the answers should show some application to the scenario):

1. As a patient-centred family doctor, how would you address the parents’ concerns regarding the vaccine? (5 marks)

Answer: Any five of the listed options below:

- Listen actively: Allocate time to genuinely hear parents’ concerns without judging, recognising their feelings and perspectives as valid.
- Build trust: Employ empathetic language and a respectful attitude to establish mutual trust, a vital element for productive conversations about vaccines.
- Ask open-ended questions to understand their reasons for hesitancy and what they value most. Listen carefully to the parents’ concerns to ensure they feel heard and that their worries are taken seriously. Also, recognise the mixed messages in the media that contribute to vaccine hesitancy.
- Validate and correct misconceptions: Carefully address and correct any misinformation using accurate and fair data while avoiding overwhelming parents with too much information at once.
- Personalise the information: Adapt your communication and educational resources, such as vaccine information statements, to the parents’ cultural background, literacy level and specific concerns.
- Emphasise benefits and risks: Discuss both the advantages of vaccines and the dangers of vaccine-preventable diseases but present the information positively by highlighting the high safety levels of vaccines.
- Reassure parents that adverse drug events are handled seriously and that there are procedures for reporting and thorough investigation.
- Reassure parents that health issues after vaccination are often coincidental and not caused by the vaccine itself. Occasionally, they might be linked to the storage, transportation or administration of the vaccine. Assure parents that the child’s swelling will be thoroughly investigated to determine its cause.
- Ensure comprehension and offer additional details on common vaccine side effects, along with leaflets or other relevant materials to support understanding.

2. Research involving children is crucial to detect possible effects of medical interventions. What are the requirements set by the research ethics committee for enrolling children in a study? (5 marks)

Answer: Any six of the listed options below:

- Children and adolescents should participate in research when their participation is scientifically essential to the research.
- Research involving children and adolescents should be properly reviewed, including by specialists in paediatric or child research.
- Children should be involved in research that respects their privacy interests. Despite their legal dependence, children have important privacy rights. Their genetic privacy may be even more crucial than that of adults with a specific genetic condition.
- Taking part would not be contrary to the best interests of the child.
- The research presents acceptable standards of risk for child participants.
- The research must take into account children’s privacy interests.
- Researchers must familiarise themselves with the legal obligations to report child abuse and neglect.
- The researcher must obtain appropriate permission, such as consent from a parent or a guardian, a substitute, or the children themselves, depending on various factors.
- Children and adolescents should be involved in research only when such research entails acceptable risks of harm.

3. The child was brought to your clinic due to swelling in the left arm. Further assessment and X-ray examination revealed a probable bone tumour in the proximal humerus, which was confirmed as an osteosarcoma by histological report. How would you approach delivering the bad news to the child’s family? (6 marks)

Answer:

The ‘SPIKES’ approach is an effective way to structure a breaking bad news consultation. One mark is awarded for each step applied to the scenario.

- Setting: The discussion should occur in a comfortable, quiet and private room in the health facility. Establish whether the patient will be participating in the meeting. A multidisciplinary team should participate to answer the direct impact on the child’s care. Arrange for a health interpreter if necessary. (1 mark)
- Perception: Establish what the child and parents already know or expect. Correct any misinformation about the child’s clinical condition. Revisit the doubt around the vaccine. (1 mark)
- Invitation: Assess the child’s and parents’ readiness to receive the results. Be attentive to the family’s culture, race, religious beliefs and socio-economic background. Encourage questions. (1 mark)
- Knowledge: Present information in manageable portions and regularly assess the child’s and parents’ understanding. Relate new information to what parents and children already know. Use visual aids or handouts to clarify the child’s clinical condition. (1 mark)
- Emotions and empathy: Recognise and respond to emotions with acceptance, empathy and concern. Use empathic responses after observing their emotional response, for example, ‘I can see how upsetting this is for you’. Use exploratory questions to clarify thoughts or feelings expressed by parents and child, for example, ‘Could you tell me what you’re worried about?’ (1 mark)
- Strategy and summary: Ask if parents and child are ready to discuss the treatment plan or next steps. Assure the family of their participation in care decisions. Plan to meet the child and parents again and inform them of the follow-up arrangements with the Orthopaedic and Oncology departments. Provide information about support services (e.g. social work, spiritual care, health psychology, support groups, community resources) (1 mark)

4. The orthopaedic surgeon has advised amputation of the arm to prevent metastasis. The child’s parents refuse the amputation. Use the four ethical principles to describe the ethical dilemma(s) arising in this case. (4 marks)

Answer:

- Respect for patient autonomy (the child) – The patient is a 14-year-old child, so she can consent to the surgical procedure if she understands what is involved (capacity), but the parents should give their assent. We are uncertain about the child’s wishes in this scenario, especially if in conflict with her parents. (1 mark)
- Justice – As a 14-year-old child, she has the right to health regardless of her parents’ decision of assent (rights-based justice). On the grounds of legal justice, the case should be reported to the magistrate to proceed with the procedure if the parents’ lack of consent obstructs the child’s rights (legal justice). (1 mark)
- Beneficence (do good) – The surgeon believes the parents are not acting in the child’s best interests. The surgeon aims to do good and to act in the patient’s best interest. Performing the surgical procedure will likely save the child’s life or improve the prognosis. However, this position conflicts with the child’s autonomy. Additionally, there is a question about how much influence the parents’ autonomy should have on the decision. (1 mark)
- Non-maleficence (do no harm) – Clearly, the surgeon’s decision (amputation of the arm) will lead to significant impairment, likely disability, psychological impact and possible social discrimination (stigma). However, this consequence is usually outweighed by the chance of dying from the cancer. (1 mark)

5. How will you address this ethical dilemma in the best interest of the child? Describe your step-by-step approach. (5 marks)

Answer: One mark for each of the steps well described:

- Step 1: Identify the moral dilemma:
  The maturity of the child, opinions of the parents, as well as the clinical circumstances, all have to be taken into consideration. The principle to bear in mind in such circumstances is that the child’s best interests are paramount. (1 mark)
- Step 2: Establish all the necessary information:
  Clinical: The child is at risk of metastasis from the osteosarcoma and death if amputation is delayed. Other treatment options may cause a lot of morbidity and are not as effective when used without surgery.
  The law: parental assent, the child’s autonomy and the doctor’s duty to protect the child – referral to a magistrate.
  Social, cultural and contextual issues from the parents’ point of view should be understood to explain their decision-making. (2 marks)
- Step 3: Analyse the information:
  Is the law clear? Parents’ refusal to assent and missing the surgical intervention is a serious threat to the child. Beneficence of the doctor needs consideration. (1 mark)
- Step 4: Formulate solution, make a recommendation:
  Educate the parents/child about the child’s clinical conditions and the consequences of delaying the surgical procedure. Provide clear information on disease progression and management options. Remind parents that the child’s rights override parental consent on legal grounds. If parents refuse to assent after counselling and health education, the doctor is obliged to seek permission from the magistrate for the surgical procedure, as the child’s rights take precedence. The doctor must act to protect the child.
- Step 5: Policy and guidelines: The Children’s Act. Clarify when a child’s consent conflicts with parental approval. (1 mark)

Total: 25 marks

### Further reading

- Mash B. Handbook of family medicine. 4th edition. Oxford: Oxford University Press, 2017; p. 540.
- Strode AE, Slack CM. Child research in South Africa: How do the new regulations help? S Afr Med J 2015;105(11):899–900. https://doi.org/10.7196/SAMJ.2015.v105i11.9838
- Consent to medical treatment in South Africa: An MPS guide [homepage on the Internet]. [cited 2025 Oct 01]. Available from: www.mps-group.org
- National Health Research Ethics Council. South African ethics in health research guidelines: Principles, processes and structures [homepage on the Internet]. 3rd ed. Pretoria: National Department of Health of the Republic of South Africa, NDoH; 2024 [cited 2025 Oct 01]; 137p. ISBN 978-0-621-52027-9. Available from: https://www.health.gov.za/nhrec-guidelines/

## Critical appraisal of research

Read the accompanying article carefully and then answer the following questions. As far as possible, use your own words. Do not copy out chunks from the article. Be guided by the allocation of marks concerning the length of your responses.

Malepe TC, Havenga Y, Mabusela PD. Barriers to family-centred care of hospitalised children at a hospital in Gauteng. Health SA Gesondheid. 2022;27:1786. https://doi.org/10.4102/hsag.v27i0.1786

1. Identify two distinct arguments made by the authors to justify the rationale for this study. (2 marks)
2. The authors state they used a ‘descriptive qualitative research design’. Critically appraise this choice in relation to their research aim. (3 marks)
3. Drawing on your understanding of qualitative methodologies, discuss whether a phenomenological approach might have been a more suitable choice for this research question. Justify your answer by referencing the differences between descriptive and interpretive phenomenology methods. (3 marks)
4. Appraise the sampling strategy used in this study. (2 marks)
5. The authors state they implemented strategies to ensure trustworthiness. Identify and critically appraise the adequacy of two specific strategies they used to enhance the study’s credibility. (4 marks)
6. Are the participants’ voices sufficiently represented, and do the conclusions logically follow from the data? Justify your answer. (2 marks)
7. A high-quality report should include a statement locating the researcher culturally or theoretically. Critically comment on whether the authors have addressed this in their methods. (2 marks)
8. Using the ‘Applicability’ component of the READER framework, describe one specific practical change you, as a family physician in a South African district hospital, could implement or advocate for, based directly on the study’s findings. (2 marks)

Total: 20 marks

### Suggested answers

1. Identify two distinct arguments made by the authors to justify the rationale for this study. (2 marks)
  Two distinct arguments made by the authors to justify the study are:
  - Despite the known benefits of family-centred care (FCC) in improving outcomes for hospitalised children, such as speeding up recovery and reducing readmissions, its implementation in practice remains limited and problematic.
  - While barriers to FCC were known to exist generally, the specific barriers within the study hospital’s paediatric wards had not yet been formally identified, as there were no policies specifically highlighting FCC principles. Exploring these barriers from the dual perspectives of nurses and caregivers was necessary to shape an optimal care environment.
2. The authors state they used a ‘descriptive qualitative research design’. Critically appraise this choice in relation to their research aim. (3 marks)
  - The authors’ stated aim was to describe the barriers to FCC by exploring nurses’ views and primary caregivers’ experiences. The choice of a descriptive qualitative research design is appropriate and congruent with this aim. The authors justified the design choice, linking it to the theoretical framework provided, namely the Theory for Health Promotion in Nursing.
  - This design is suitable for research that seeks to provide a rich, detailed account of a phenomenon as it is experienced in a particular context. The study aimed to explore practical issues by collecting ‘dynamic and holistic views from participants’ to inform improvements in care.
  - When a study focuses on people’s ‘ideas, beliefs, opinions or perceptions’ rather than their lived experience, the design is often an ‘exploratory descriptive qualitative study’, which aligns with the authors’ focus on ‘nurses’ views’.
3. Drawing on your understanding of qualitative methodologies, discuss whether a phenomenological approach might have been a more suitable choice for this research question. Justify your answer by referencing the differences between descriptive and interpretive phenomenology methods. (3 marks)
  - While the chosen design was appropriate, a phenomenological approach could have been a more suitable and methodologically specific choice, particularly for exploring the caregivers’ experiences. Phenomenology is concerned explicitly with understanding the ‘lived experience’ of a phenomenon. The interview question for caregivers – ‘Tell me about your experience of having a child in hospital’ – is a classic opening for a phenomenological inquiry.
  - Had the researchers used descriptive phenomenology, the aim would have been to describe the universal ‘essence’ of experiencing barriers to FCC. This would have required the researchers to ‘bracket’ or set aside their own assumptions to focus purely on the participants’ conscious experience.
  - An interpretive phenomenological approach might have been even more suitable. This approach goes beyond description to explore the meaning of the experience for participants within their specific social context. Given that the study’s findings centred on interpersonal issues such as mistrust and poor communication, interpretive phenomenology would have enabled a deeper exploration of what these barriers mean to caregivers and nurses within the context of a South African public hospital.
4. Appraise the sampling strategy used in this study. (2 marks)
  - The study used purposive sampling to select nurses and primary caregivers who met specific inclusion criteria. This strategy is suitable for qualitative research, as it aims to select ‘information-rich’ participants who can provide in-depth insights into the phenomenon.
  - A significant strength was the inclusion of two different populations (nurses and caregivers), which allowed for a more comprehensive view of the barriers. A weakness is that findings from purposive sampling are not intended to be generalisable to a broader population.
5. The authors state they implemented strategies to ensure trustworthiness. Identify and critically appraise the adequacy of two specific strategies they used to enhance the study’s credibility. (4 marks)
  The authors identify two strategies used to enhance credibility:
  - Strategy 1: Participant triangulation. The study included two distinct groups – nurses and primary caregivers. This is an adequate and strong strategy, as collecting different perspectives on the same phenomenon allows for a more comprehensive and robust understanding of the barriers than if only one group had been interviewed.
  - Strategy 2: Member checking. The researchers performed this at the end of each interview by summarising the content to confirm their understanding with the participant. This is an inadequate, or at least limited, implementation of this strategy. While helpful, this method is less rigorous than when participants are asked to validate the fundamental statements or themes after the initial analysis is complete, providing a better opportunity to confirm that their views were accurately interpreted.
6. Are the participants’ voices sufficiently represented, and do the conclusions logically follow from the data? Justify your answer. (2 marks)
  - Yes, this criterion is met to a high standard. The authors ensure participants’ voices are represented by providing numerous, verbatim quotes from both ‘Primary caregiver’ and ‘Nurse’ to support every theme and sub-category in the findings section.
  - This paper provides a clear audit trail (‘well-kept documentation and transparency in the methodology, data analysis, and conclusions’), demonstrating that the conclusions are grounded in the data and logically flow from the analysis of the participants’ own words.
7. A high-quality report should include a statement locating the researcher culturally or theoretically. Critically comment on whether the authors have addressed this in their methods. (2 marks)
  - The article fails to meet this criterion. A high-quality qualitative report should include a statement locating the researcher and addressing their influence on the research.
  - The authors do not provide a reflexivity statement in their methods section. They do not discuss their own backgrounds, potential biases or assumptions regarding FCC, nor do they detail how their presence might have influenced the research process or thematic analysis of the participants’ responses.
8. Using the ‘Applicability’ component of the READER framework, describe one specific practical change you, as a family physician in a South African district hospital, could implement or advocate for, based directly on the study’s findings. (2 marks)
  As a family physician in a South African district hospital, I would adopt a practical approach to care and advocate for initiating a clinical governance project to review and revise the paediatric ward’s policies on visitation and parental accommodation.
  - The study’s key finding directly informs this action, which is that rigid ward policies were a significant barrier to FCC for both nurses and caregivers.
  - Implementation would involve forming a working group including hospital management, ward staff and primary caregivers to co-design more flexible, family-centred policies. This directly aligns with the study’s specific recommendation to include ‘the voice of the community’ in policy revisions.

### Further reading

- Pather M. Evidence-based family medicine. In: Mash B, editor. Handbook of family medicine. 4th ed. Cape Town: Oxford University Press, 2017; p. 430–453.
- JBI. Critical appraisal tools [homepage on the Internet]. 2025 [cited 2025 Sep 25]. Available from: https://jbi.global/critical-appraisal-tools
- Mash R, Ajudua F, Malope S, Kaura D. Phenomenology for primary care researchers. Afr J Prim Health Care Fam Med. 2025;17(2):4946. https://doi.org/10.4102/phcfm.v17i2.4946

## Objectively Structured Clinical Examination station scenario EPA 5: Managing children requiring inpatient care and procedures

### Objective of station

**FIGURE 1:**
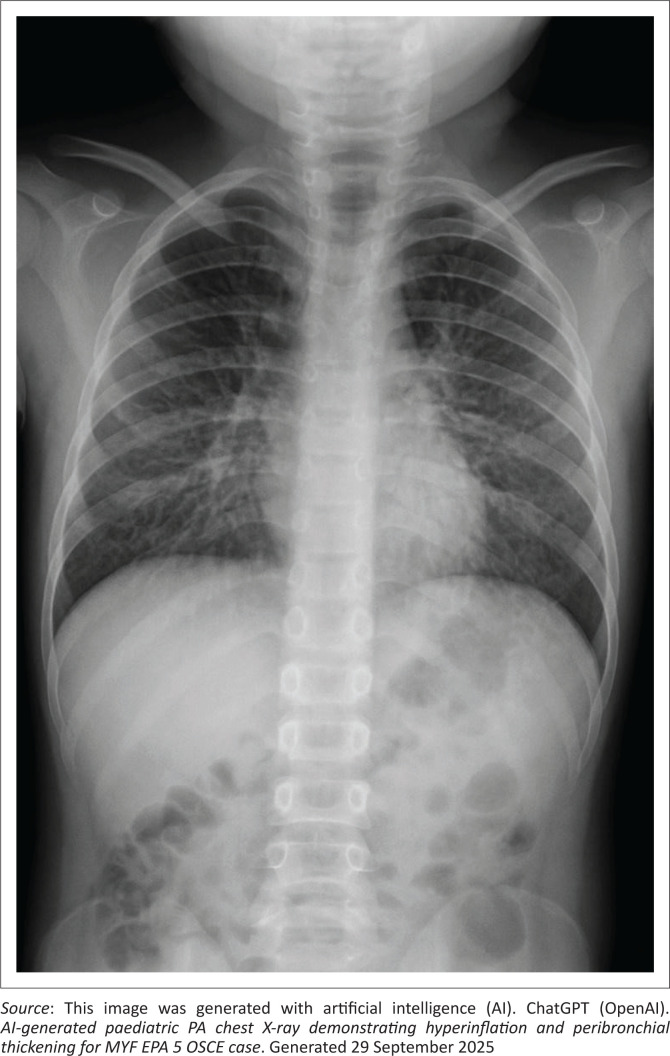
Posteroanterior (PA) chest X-ray.

**FIGURE 2:**
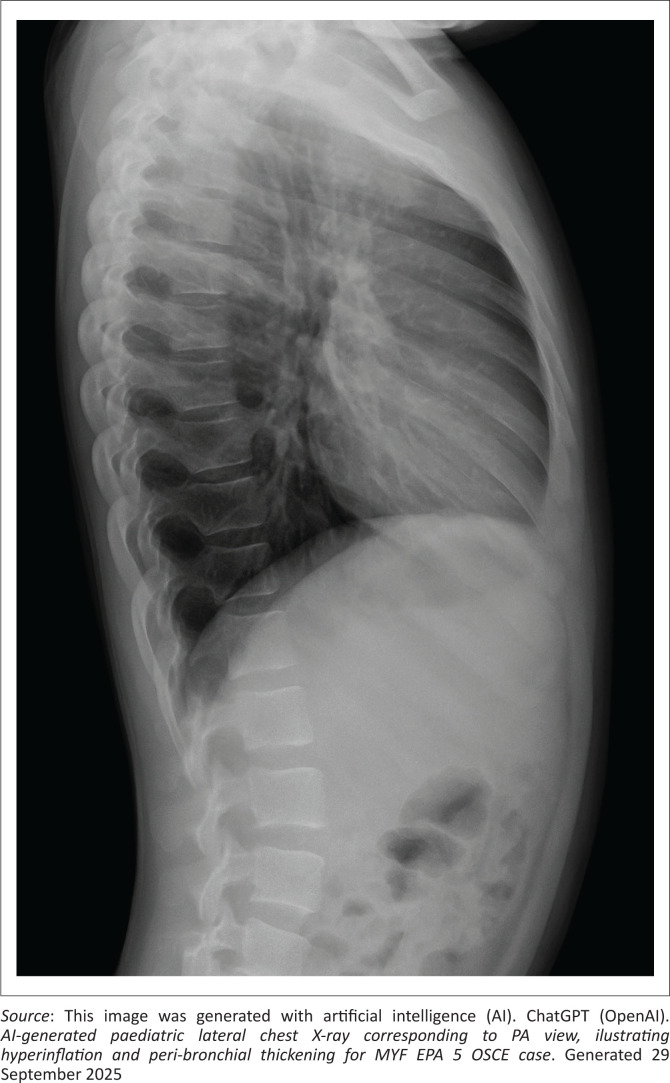
Lateral chest X-ray.

This station tests the candidate’s ability to conduct an online consultation with the parent of a child requiring inpatient care (see Figures 1 and 2).

### Type of station

Integrated consultation.

### Role players

Mother of a 6-year-old child.

### Instructions for the candidate

You are the family physician on duty in the *paediatric ward at a district hospital*.

A 6-year-old boy was admitted earlier today with breathing difficulties. You are asked to review the child and speak with his mother.

Your task:

- Please consult with the mother to obtain relevant information and explain the situation to her.
- Discuss your comprehensive assessment with her and outline your immediate and ongoing management plan.

### Instructions for the examiner

- This is an integrated consultation station in which the candidate has 20 min.
- See Table 1 for the marking sheet familiarise yourself with the assessor guidelines, which detail the required responses expected from the candidate.
- No marks are allocated. In the marking sheet, tick off one of the three responses for each of the competencies listed. Make sure you are clear on what the criteria are for judging a candidate’s competence in each area.

**TABLE 1:** Marking sheet for consultation station.

Competencies	Candidate’s rating		
	Not competent	Competent	Good
Establishes and maintains a good doctor–patient relationship	-	-	-
Gathering information: history/examination/investigations	-	-	-
Clinical reasoning	-	-	-
Explanation and planning	-	-	-
Management	-	-	-

### Guidance for examiners

The station assesses whether the candidate can:

- Conduct a consultation with the parent of a child requiring inpatient care.
- Demonstrate empathy and effective communication with a distressed caregiver.
- Gather relevant biomedical and psychosocial information through focused questions.
- Apply clinical reasoning to diagnose and prioritise management of severe acute asthma.
- Explain the condition and outline a safe, evidence-based management plan.
- Integrate long-term control, caregiver education and continuity of care.

A working definition of competent performance: the candidate effectively completes the task within the allotted time, in a manner that maintains patient safety, even though the execution may not be efficient and well structured.

■ *Not competent:* patient safety is compromised (including ethically and legally), or the task is not completed.
■ *Competent*: the task is completed safely and effectively.
■ *Good*: in addition to displaying competence, the task is completed efficiently and in an empathic, patient-centred manner (acknowledges patient’s ideas, beliefs, expectations, concerns/fears).

## Establishes and maintains a good clinician–intern–patient relationship

The competent candidate shows genuine respect, rapport and empathy; establishes trust and attends to the mother’s anxiety after introducing themselves and determining the reason for the consultation. Shows basic empathy and acknowledges the patient’s distress. Maintains confidentiality and ensures that the online space is safe.

In addition to the above, the good candidate acts within the ethical framework (respects autonomy, justice, non-maleficence and beneficence) in acknowledging the mother’s concerns, builds strong rapport through active listening, non-verbal skills and empathic response and demonstrates cultural humility and shared decision-making, together with shared responsibility for asthma control.

## Gathering information

The competent candidate elicits a history of acute illness and prior asthma episodes; asks about correct inhaler use (poor response to salbutamol), adherence (only reliever, no controller) and clinic visits; screens for allergies and triggers; explores social and environmental risk factors (smoke, pets, housing) and identifies barriers to long-term management (access, finances, knowledge and poor follow-up and hospital admissions).

In addition to the above, the good candidate explores the caregiver’s beliefs and understanding of asthma, identifies caregiver stress and family impact and explores social/schooling/sleep impact on the child. Atopic history and family history of atopy should be explored.

## Clinical reasoning

The competent candidate recognises **acute severe asthm**a with hypoxia; integrates acute episode with chronic poor control, not on inhaled corticosteroids;considers allergen triggers and environmental exposures and rules out pneumonia/TB, but no supporting evidence.

In addition to the above, the good candidate anticipates risk of deterioration (status asthmaticus) and balances immediate treatment with long-term prevention.

## Explaining and planning

The competent candidate explains severe asthma clearly and calmly, utlines immediate management (oxygen, nebulisers, steroids), explores concept of stepping up and stepping down treatment based on symptoms and control, provides reassurance and clear next steps, gives a holistic explanation (acute vs. chronic aspects), uses visual aids (explains peak flow chart, CXR report) and counsels on prevention (ICS use, trigger avoidance).

In addition to the above, the good candidate negotiates a realistic care plan considering social constraints and educates on inhaler technique, red flags and follow-up plan.

## Management

The competent candidate states immediate inpatient treatment:

- Oxygen (sat > 94%)
- Nebulised salbutamol + ipratropium
- Corticosteroids (oral/IV)
- IV access and fluids if poor intake
- Monitoring plan: vitals, peak expiratory flow rate (PEFR) and reassessment.

Full inpatient management plan:

- Escalation pathway (IV MgSO_4_/aminophylline/adrenaline, tertiary referral if failing)
- Nutritional support and growth monitoring
- Initiate controller therapy (ICS) before discharge
- Inhaler technique education
- Asthma action plan for the mother and the school
- Manage comorbid conditions such as allergic rhinitis for improved outcomes overall

**FIGURE 3:**
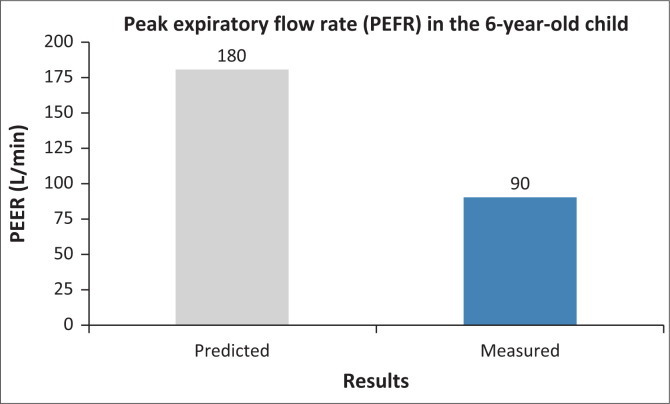
PEFR showing predicted and measured results.

In addition to the above, the good candidate provides social work referral (grants, CHW follow-up, smoking cessation support). Arrange continuity: clinic follow-up, education on adherence and trigger avoidance.

## Role play: Instructions for mother

### Child’s details

- Name: Mfundo, 6-years-old, weight 20 kg.
- School: Grade 1.

### Presenting illness

- Acute shortness of breath, wheezing since last night.
- Mother tried his ‘blue pump’ (salbutamol inhaler) at home, but it did not help.
- History of recurrent wheezing since the age of 3 years, three hospital admissions last year and one ICU admission.

### Background

- Diagnosed with asthma at the age of 4 years, only on PRN salbutamol, not on regular inhaled corticosteroid.
- Allergic rhinitis and eczema.
- Immunisations up to date.
- No TB contacts. No HIV in family.

### Social context

- Lives in a shack with mother, grandmother and two siblings.
- Mother smokes cigarettes inside the house (defensive if challenged).
- Has a pet cat (sleeps in the same bed as the child).
- Mother is unemployed, on social grant.
- The school has complained of frequent absenteeism.

**FIGURE 4:**
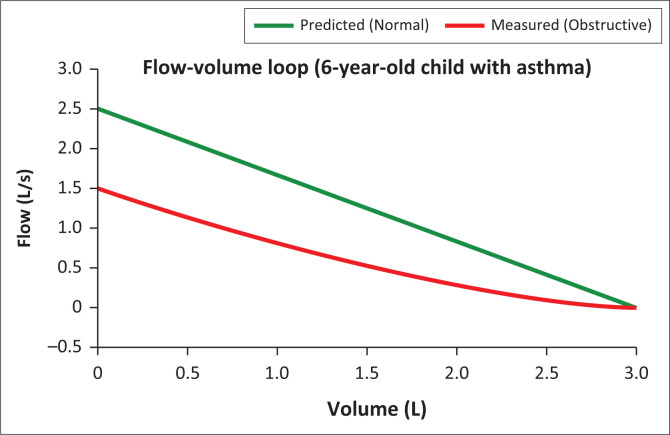
Flow volume loop for the 6-year-old child.

### Mother’s attitude (to role-play)

- Very anxious: ‘Doctor, is he going to stop breathing?’
- Asks if the child has pneumonia or TB.
- Doesn’t understand why the inhaler sometimes works and sometimes doesn’t.
- Wants to know if asthma can be cured.
- Guilty but defensive about smoking.
- Opens up about financial struggles if the doctor shows empathy.

### General role-playing notes:

- You are *the mother of Mfundo*, a 6-year-old boy, 20 kg, Grade 1, admitted this morning with severe asthma.
- You are on a *Zoom call* with the candidate (doctor).
- The child is not on camera – you are speaking on his behalf.
- Your goal is to *test the candidate’s ability to communicate, counsel and manage an inpatient case via a parent*.
- Adjust your emotional tone depending on how well the candidate engages with you.

### Emotional baseline

- *Start anxious and fearful*: you are worried your child might die.
- If the candidate is empathetic → you relax, become cooperative and open.
- If the candidate is cold, dismissive or judgemental → you become defensive and withdrawn.

### Opening line (when doctor greets you)

‘Doctor, thank you for seeing me. Mfundo is really struggling to breathe… is he going to stop breathing?’

### Information to provide (if candidate asks)

Presenting illness – only if asked:

- ‘He started coughing and wheezing last night’.
- ‘I gave him his blue pump (inhaler), but it didn’t help this time’.
- ‘This morning he was worse, so I rushed him to the hospital’.

Medical history – only if asked:

- ‘He has had chest problems since he was 3-years-old’.
- ‘He’s been admitted three times in the last year – once he was even in ICU’.
- ‘The clinic told me it’s asthma’.
- ‘He only uses the blue pump when he’s bad. I don’t know about the other pumps they mentioned’.
- ‘Has allergic rhinitis and eczema’ sometimes
- Immunisations up to date
- No TB exposure, no HIV in the family

Social and/or family context – only if asked:

- Lives with mother, grandmother and siblings in a shack.
- ‘I smoke, but only at night, in the house. It doesn’t affect him, does it?’ (say this defensively).
- ‘We have a cat, he loves it and sleeps with it’.
- ‘I don’t always have money to go to the clinic for pumps’.
- ‘His school keeps complaining about him missing days’.
- ‘I am unemployed and rely on a social grant only’
- ‘School often complains of absenteeism’

Caregiver concerns – only if asked:

- ‘Doctor, is this TB or pneumonia?’
- ‘Why doesn’t the inhaler always work?’
- ‘Will my child grow out of this? Can it be cured?’
- ‘Will he need to go to Johannesburg hospital again?’

### Emotional cues

- If the candidate **explains clearly and shows empathy**:
  Say: ‘That makes sense, doctor. I didn’t know asthma could be controlled. I just thought it happened randomly’.
  Become calmer and more cooperative.
- If the candidate *judges you about smoking*:
  Respond defensively: ‘I can’t just stop, doctor. I’m already stressed, and he sleeps fine most nights’.
- If the candidate *addresses smoking sensitively*:
  Respond thoughtfully: ‘I didn’t realise it could make things worse. Maybe I can try smoking outside instead’.
- If the candidate **reassures you that the hospital has a plan**:
  Say: ‘Thank you, doctor, I feel a bit better knowing he won’t die tonight’.

### Specific questions to ask the candidate

(Use at different points if the candidate hasn’t addressed them)

- ‘Why does this keep happening?’
- ‘Can asthma be cured?’
- ‘Why does the pump sometimes not work?’
- ‘What can I do at home to stop this from happening again?’
- ‘When should I bring him back if he gets sick again?’

### Key ‘triggers’ for candidate competence

- If they *ask about social history* → provide information about smoking, cat and poor finances.
- If they *ask about adherence/medication* → mention only using the blue pump, no steroid pump.
- If they *ask about clinic follow-up* → mention difficulty with transport and running out of pumps.
- If they *acknowledge your fear* → you calm down.
- If they *explain clearly what asthma is* → you express relief and interest.

### Closing line

If the candidate has explained well:

- ‘Thank you, doctor. I understand better now and I’ll try to do what you say. I just want Mfundo to get better’.
- If the candidate was unclear or rushed:
- ‘I still don’t really understand what’s wrong with him. I’m very worried’.

## Examination findings and investigations

### Current vital signs

- Temperature 37.2°C
- HR 150 bpm
- RR 48/min
- O_2_ sat 89% on room air → 93% with facemask O_2_
- PEFR 90 L/min (expected ~180)

### Exam

- Sitting upright, speaking in short phrases, with subcostal recession.
- Widespread expiratory wheeze, poor air entry.
- No cyanosis, no focal chest signs.
- Capillary refill < 2 s.

### CXR report

- Hyperinflated lungs, peribronchial thickening, no consolidation.

### Full blood count

- Mild eosinophilia.
- PEFR

